# Optimization of Polyhydroxybutyrate Production by Amazonian Microalga *Stigeoclonium* sp. B23

**DOI:** 10.3390/biom10121628

**Published:** 2020-12-03

**Authors:** Murilo Moraes Mourão, Diana Gomes Gradíssimo, Agenor Valadares Santos, Maria Paula Cruz Schneider, Silvia Maria Mathes Faustino, Vitor Vasconcelos, Luciana Pereira Xavier

**Affiliations:** 1 Laboratory of Biotechnology of Enzymes and Biotransformations, Institute of Biological Sciences, Federal University of Pará, 01 Augusto Corrêa Street, 66075-110 Belém, Pará, Brazil; dianagradissimo@gmail.com (D.G.G.); luxavier@gmail.com (L.P.X.); 2 Genomics and Systems Biology Center, Federal University of Pará, 01 Augusto Corrêa Street, 66075-110 Belém, Pará, Brazil; mariapaulacruzschneider@gmail.com; 3 Laboratory of Algae Cultivation and Bioprospecting, Pharmacy Coordination, Federal University of Amapá, Marco Zero do Equador Campus, Juscelino Kubitschek Highway, Km 2, 68903-419 Macapá, Amapá, Brazil; silviamathes@unifap.br; 4 Interdisciplinary Center of Marine and Environmental Research, University of Porto, General Norton de Matos Av., 4450-208 Porto, Portugal; vmvascon@fc.up.pt; 5 Department of Biology, Faculty of Sciences, University of Porto, Campo Alegre Street, 4069-007 Porto, Portugal

**Keywords:** Amazon, microalgae, Stigeoclonium, optimization, polyhydroxybutyrate

## Abstract

The present work established the optimization and production of biodegradable thermoplastic polyhydroxybutyrate (PHB) from Amazonian microalga *Stigeoclonium* sp. B23. The optimization was performed in eight different growth media conditions of *Stigeoclonium* sp. B23, supplemented with sodium acetate and sodium bicarbonate and total deprivation of sodium nitrate. B23 was stained with Nile Red, and PHB was extracted and quantified by correlating the amount of fluorescence and biopolymer concentration through spectrofluorimetry and spectrophotometry, respectively. Our results detected the production of PHB in *Stigeoclonium* sp. B23 and in all modified media. Treatment with increased acetate and bicarbonate and without nitrate gave the highest concentration of PHB, while the treatment with only acetate gave the lowest among supplemented media. Our results showed a great potential of *Stigeoclonium* sp. B23, the first Amazonian microalga reported on PHB production. The microalga was isolated from a poorly explored and investigated region and proved to be productive when compared to other cyanobacterial and bacterial species. Additionally, microalga biomass changes due to the nutritional conditions and, reversely, biopolymer is well-synthetized. This great potential could lead to the pursuit of new Amazonian microalgae species in the search for alternative polyesters.

## 1. Introduction

The Earth has been facing the need for renewable energies for a long time, including biofuels and plastics made from microalgae and cyanobacteria from natural sources. Besides the depletion of petroleum sources and the consequences of its use on both the environment and on living things, many countries have been investing in ecologically sustainable solutions, focussing especially on renewable energies, e.g., biodegradable plastics that are able to mitigate the needs of global demand [1]. Polyhydroxybutyrates (PHB) are biopolymers, a type of polyesters produced by many bacteria species, including cyanobacteria [2]. They are stored as energy carbon reserves in the form of insoluble intracellular cytoplasmic granules, and their functions have been documented for osmotic pressure resistance processes, cell desiccation defense, and ultraviolet rays [1,2,3]. PHB has drawn significant attention to the plastics industry, and its use could contribute to the convergence of various research methods and many industries by means of a circular economy in order to increase production of PHB, which can provide an acceptable alternative with less environmental impacts due to its biodegradable thermoplastic properties [4]. PHBs are also elastomers and can potentially be used in aqueous solution, when they are resistant and have similar physicochemical properties to different petrochemical plastics such as polyethylene and polypropylene [2,3]. PHB can be useful in several industrial fields, most commonly in the packing industry and for bags to support the recycling process and decreases environmental problems caused by waste accumulation [5]. Additionally, the biopolymers can be useful in the pharmaceutical industry as biodegradable carrier biomaterials for drug delivery capsules, and they can also be used for replacement therapy of bones and blood vessels and as scaffolds for tissue engineering [6].

Currently, more than 100 species of cyanobacteria have been reported as being capable of synthesizing and storing the biopolymer, with about 70% of them able to synthesize and store PHB in a range of 0.004% to 6% of dry mass of the biopolymer under photoautotrophic conditions [7]. Studies about the identification and production of PHB in oleaginous filamentous microalgae are recent and promising, offering a suitable alternative [8]. Recently, *Chlorella fusca* LEB 111 was induced by supplementation with xylose under 6 h of light exposure with an intensity of 28 μmol.photons/m^2^ s, and it obtained 17.4% (*m/m*) of polyhydroxyalkanote (PHA) [9]. The maximum amount of dry weight of PHB in a transgenic lineage was found in *Chlamydomonas reinhardtii* microalga cultivated with Tris-Acetate-Phosphate (TPS) with 6 μg/g [10]. In a more in-depth study, the maximum dry weight yield of PHB (247 mg/mL) obtained by *Botryococcus braunii* Kütz, which represents 60% of its dry weight, was obtained with wastewater cultivation from sewage treatment [7].

In bacteria and cyanobacteria, different forms of PHB are known and are well-studied, but not much is known about PHB production in eukaryotic microalgae. In this group, PHB is synthesized under limited conditions of one or two essential nutrients, such as nitrogen [11], phosphorus [12], magnesium [13] or oxygen [14], to induce accumulation and storage. Supplementation with carbon sources such as atmospheric CO_2_ [1], sugarcane bagasse [15], sodium bicarbonate [16], sodium acetate [17], and many others can provide maximum yields of PHB productivity. The maximum amount of PHB can reach about 10 wt % of dry cell weight under nitrogen deprivation and supplemented with sodium acetate [17,18,19]. These nutrient deprivations and supplementations describe important advantages for the production of secondary metabolites [20].

PHB synthesis can occur during and after the exponential process, even in the stationary growth phase, depending on the species [21]. The quantification and detection of PHB granules in microorganisms have been performed using fluorescent dyes [22,23,24,25,26,27]. The quantification of PHB requires solvents for extraction and consequently can be read by gravimetric methods or by spectrophotometry, resulting in time spent, thus obtaining low coverage of the analyzes [25].

The Amazon biome is currently considered to be one of the greatest resources available in Brazil. It is a mega-diverse ecosystem, providing infinite and valuable resources for the region and the world. Therefore, the great potential of Amazonian biodiversity in the production of polyhydroxybutyrates by microorganisms is undeniable. However, even with this great potential, there are no reports regarding the production of PHB from cyanobacteria or microalgae from the Amazon Rainforest. In addition, it is estimated that bioplastics production will reach about US $6.1 million in 2021 in the global market, but there are problems regarding the cost of PHB production, the demand for new techniques for reducing the production process, and optimization of several parameters to obtain higher biopolymer yields [4,7]. Some species of the genus Stigeoclonium have been described as potential producers of lipids used in biofuels characterization, but there are no reports on the production of PHB by this genus [28]. Thus, this work aimed to detect, extract, and quantify PHB produced by the eukaryotic and oleaginous green microalga *Stigeoclonium* sp. B23, under nutritional stress by nitrogen deprivation, and also evaluate the PHB production for 30 days. Our results suggest that *Stigeoclonium* sp. B23 is a good candidate for the production of polyhydroxybutyrate using small quantities of carbon sources as the main substrates for accumulation of the biopolymer.

## 2. Materials and Methods

### 2.1. Characterization and Identification of B23 Strain

The morphological and physiological characteristics of the strain B23 were investigated according to morphological analysis proposed by Vischer [29].

### 2.2. 18S rRNA Gene Sequence Analysis

The genomic DNA was extracted from the B23 strain cultured using the PowerPlant^®^ Pro DNA Isolation kit (MO BIO Laboratories, Inc.). From the genomic DNA, 18S rRNA sequences were amplified by PCR using primers SR-8 (GGATTGACAGATTGAGAGCT) and INT-5R (AGGTGGGAGGGTTTAATGAA) [30]. After amplification, the PCR product was sequenced by the method of Sanger [31] using an ABI 3730 DNA Analyzer sequencer (Applied Biosystems). The DNA fragments were aligned and curated in BioEdit, and the complete 18S RNA sequences of the strain were compared to those of GenBank via Basic Local Alignment Search Tool (BLAST) to confirm identification based on morphology.

### 2.3. Media and Growth Conditions

The B23 strain (HAMAB accession number 019132), native of the state of Amapá (Brazil), was isolated from a puddle of water and used in the present study. The strain was provided by Genomics and Systems Biology Center (CGBS) of the Federal University of Pará (Belém, Brazil).

Microalga was grown in BG-11: 1.5 g/L NaNO_3_, 0.04 g/L K_2_HPO_4_, 0.075 g/L MgSO_4_.7H_2_O, 0.036 g/L CaCl_2_.2H_2_O, 0.006 g/L citric acid, 0.006 g/L ferric ammonium citrate, 0.001 g/L Na_2_EDTA, 0.02 g/L sodium carbonate; micronutrients: 2.86 g/L H_3_BO_3_, 1.81 g/L MnCl_2_.4H_2_O, 0.222 g/L ZnSO_4_.7H_2_O, 0.39 g/L Na_2_Mo_4_.2H_2_O, 0.079 g/L CuSO_4_.5H_2_O, 0.049 g/L Co(NO_3_)_2_.6H_2_O [32]. For PHB synthesis, the cultivation was supplemented with 0.82 g/L and 0.42 g/L of sodium acetate and sodium bicarbonate, respectively. The cultures were incubated for 30 days at a controlled temperature of 25 °C for a photoperiod of 12 h dark/12 h light, with a pH of 7. These parameters of cultivation are typical of *Stigeoclonium* sp. [28]. Resuspension of the culture was performed in eight modified BG-11 media.

### 2.4. Chlorophyll a Content

For the concentration of chlorophyll *a*, 1 mL of the culture was collected every 2 or 3 days for a period of 30 days (13 harvests in total). Chlorophyll *a* concentration was performed in triplicate and periodically every 2 days for 30 days. During the cultivation, aliquots were taken and standardized on culture flasks prior to obtaining uniform samples. This consisted of adding to the centrifuged pellets 1 mL methanol (9:1). The determination of the concentration of chlorophyll *a* was monitored by variations in optical densities, with absorbance of methanolic extract at a 663 nm wavelength by spectrophotometry and pure methanol as blank [33]. The following equation was used to calculate the concentration of chlorophyll *a*: C (µg/mL) = OD_663_ × 12.7; where, C (μg/mL) corresponds to the concentration of chlorophyll *a*; OD_663_ is the absorbance of the methanolic extract at an optical density of 663 nm; and 12.7 is the absorptivity coefficient of chlorophyll *a* extracted with methanol [34].

### 2.5. Fluorescent Microscopy

PHB granules were visualized for 12 days of cultivation of *Stigeoclonium* sp. B23 under fluorescence using Nile Red dye (Sigma–Aldrich, St. Louis, MO, USA). It consisted of 0.5 mL suspension of each culture centrifuged at 14,000 rpm for 20 min, and the pellets were re-suspended in 1 mL of deionized water. Then, 20 μL of Nile Red was added to the suspension (80 μg/mL in DMSO). The solution was washed again in deionized water and PHB was visualized on NIKON H550L fluorescence microscopy (NIKON, Japan) under excitation and emission wavelengths of 535 nm and 610 nm, respectively [24].

### 2.6. Quantification of PHB Fluorescence

For the quantification of PHB fluorescence, every 2 or 3 days for a period of 30 days (13 harvests in total), 1 mL of each modified culture medium was stained with 40 μL of Nile Red (80 μg/mL in DMSO), and the fluorescence was detected on a spectrofluorimeter Victor X3 (PerkinElmer, Waltham, MA, USA) with excitation and emission wavelengths of 535 nm and 610 nm, respectively [24,25,26].

### 2.7. PHB Extraction

The PHB was extracted from 60 mg of the lyophilized sample of the microalgae, adding 5 mL of sodium hypochlorite to the biomass and incubating for 2 h in a water bath at 30 °C. After homogenizing at 5000 rpm for 15 min, the pellet was washed with 0.5 mL deionized water and 0.5 mL of a solution of acetone and ethyl alcohol. The pellet was dissolved by adding 5 mL of warm chloroform to extract PHB [35,36,37].

### 2.8. Quantification of PHB Content

The PHB/chloroform solution was evaporated in a petri dish at room temperature. For the quantification of PHB, 10 mL of 100% sulfuric acid was added to the freshly extracted PHB, and the solution was heated for 10 min at 100 °C in a water bath. After the solution cooled, 1 mL was transferred to a quartz cuvette for spectrophotometer analysis with absorbance at 235 nm and pure sulfuric acid as blank. The absorbance spectrum curve in the 200–300 nm wavelength range was measured at 235 nm to confirm PHB occurrence. Crotonic acid quantity was calculated by the molar extinction coefficient 1.55 × 10^4^. A standard curve of crotonic acid was assayed in a range between 2.5 to 20 μg/mL and was finally calculated to have PHB. The values were indicated by means of the three analytical readings [35,36].

### 2.9. Statistical Analysis

Mean and standard errors of the 13 harvest days of all treatments were calculated and carried out in triplicate. Two-way ANOVA was used to test for differences in PHB fluorescence and PHB among the culture conditions by GraphPad Prism 6.01. Tukey’s multiple comparisons test was used to identify statistically significant pairwise differences. Statistical significance level was *p* = 0.05 overall.

## 3. Results

### 3.1. Morphological and Molecular Identification of Strain B23

**Figure 1:**
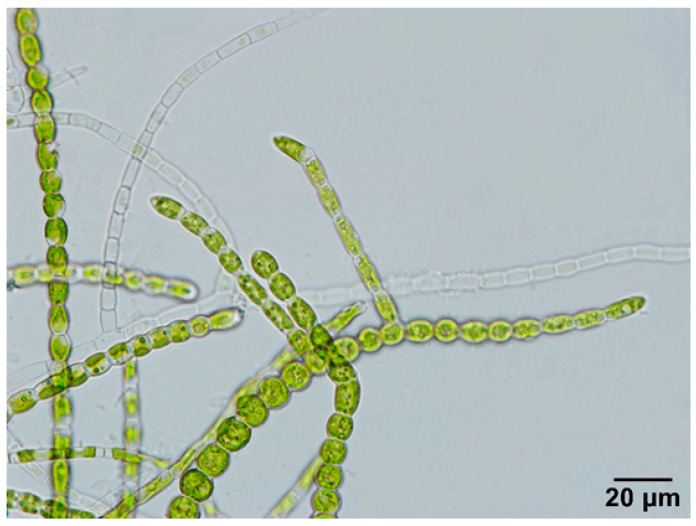
Optical image of *Stigeoclonium* sp. B23.

Morphologically, the B23 strain belongs to the genus Stigeoclonium (Figure 1). This strain has a stem composed of heterotrichius tufts, green in color, a well-developed erect system, and a reduced prostrate system composed of short rhizoids at the base of the erect filaments. Additionally, filaments are not embedded in mucilage, and each cell contains a single green chloroplast. The main axis cells are cylindrical with a diameter of 11–14.5 μm, a length of 18.5–62.8 μm, opposite or alternating branching, a presence of draparnaldia form branches, 6–8 μm lateral branch cells, and are 10–30 μm in length.

Subsequently, for molecular identification, BLAST of the 18S gene resulted in 500 nucleotides (Figure S1), determined the genus of isolate B23 as being Stigeoclonium, and was not able to identify precisely the species. However, high coverages (>85%) and identities (>90%) were obtained with 25 species belonging to the genus Stigeoclonium. For example, National Center for Biotechnology Information (NCBI) database alignments showed 100% similarity with the 18S rRNA gene sequence of *Stigeoclonium nanum* (HF920645), 99.8% with *Stigeoclonium* sp. JB10 strain (KF791547), 99.8% similarity with *Stigeoclonium tenue* strain CCAC 3492 B (HF920647), 99.6% with *Stigeoclonium* sp. CCAC 1901 B (HF920646), 99.4% with *Stigeoclonium subsecundum* strain UTEX 1574 (HF920644), and 99.2% with *Stigeoclonium farctum* CCAP: 477/10A. According to a comprehensive bibliographic study, these reference strains, recorded in several environments, did not exhibit PHB storage. This is the first strain of the Brazilian Amazon that has been shown to alter its accumulation of PHB under various growth conditions.

### 3.2. Growth of Stigeoclonium sp. B23

**Figure 2:**
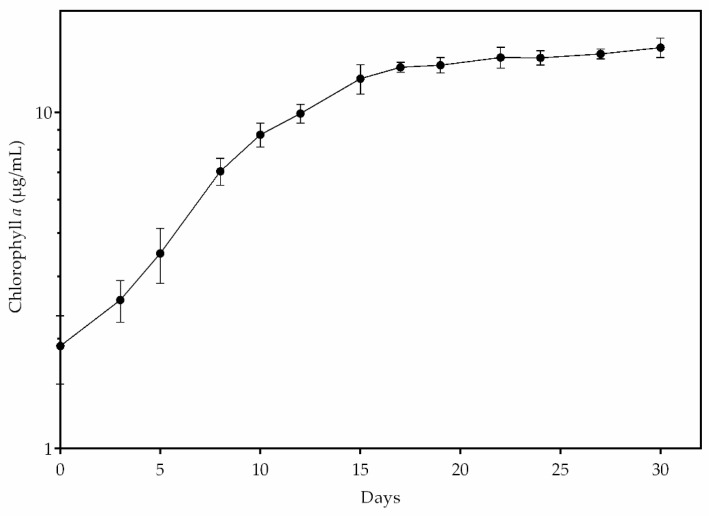
Growth of *Stigeoclonium* sp. B23 in standard medium based on the amount of chlorophyll *a* (µg/mL) at each harvest for 30 days. Data are the mean ± SD of three replicates.

A growth curve of *Stigeoclonium* sp. B23, based on the chlorophyll *a* quantity, was performed in BG-11 standard medium in order to analyze how microalga displays its pigment development, until it reached the stationary phase (Figure 2). An exponential increase was observed until day 15. From this day on, microalga began to reduce its growth and stayed in the stationary process for the next 15 days. During the experiment, it did not decrease.

### 3.3. Growth of Stigeoclonium sp. B23

**Figure 3:**
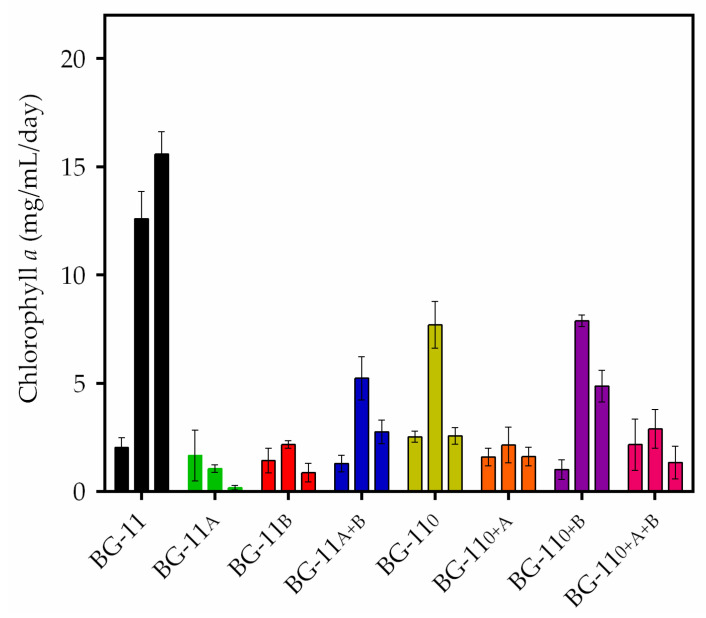
Chlorophyll *a* concentration (mg/mL/day) of the *Stigeoclonium* sp. B23 in eight culture conditions. Three time points were plotted in each cultivation: the left bar corresponds to day 0 of the cultivation, the middle bar corresponds to day 15, and the right bar to day 30. Data are the mean ± SD of three replicates.

In order to confirm the variation of chlorophyll *a* as an indirect method of measurement and the viability of the strain among the 8 modified cultures (Table 1), during 30 days of cultivation, the pigment was quantified (Figure 3). The amount of chlorophyll *a* at day 0 corresponds to the first day of cultivation, day 15 to the beginning of the stationary phase, and day 30 corresponds to the end of the stationary phase, according to the growth curve of Figure 2 of standard medium. Measurements of chlorophyll *a* were quantified every two days. Figure 3 shows the variation of pigment caused by the absorption of nutrients and the stress imposed by nitrogen deprivation along with sodium acetate and sodium bicarbonate supplementation. The maximum yield of chlorophyll *a* was 14.9 ± 0.5 μg/mL at day 27 in standard media. The amount of chlorophyll *a* corresponded to a 6.4-fold increase (approximately 86.5%) at day 27 when compared to day 0. However, it was not possible to obtain a high volume of biomass in other procedures, except for acetate and bicarbonate, without the addition of nitrate and nitrate to bicarbonate treatment on day 15.

### 3.4. PHB Detection by Fluorescence

**Figure 4:**
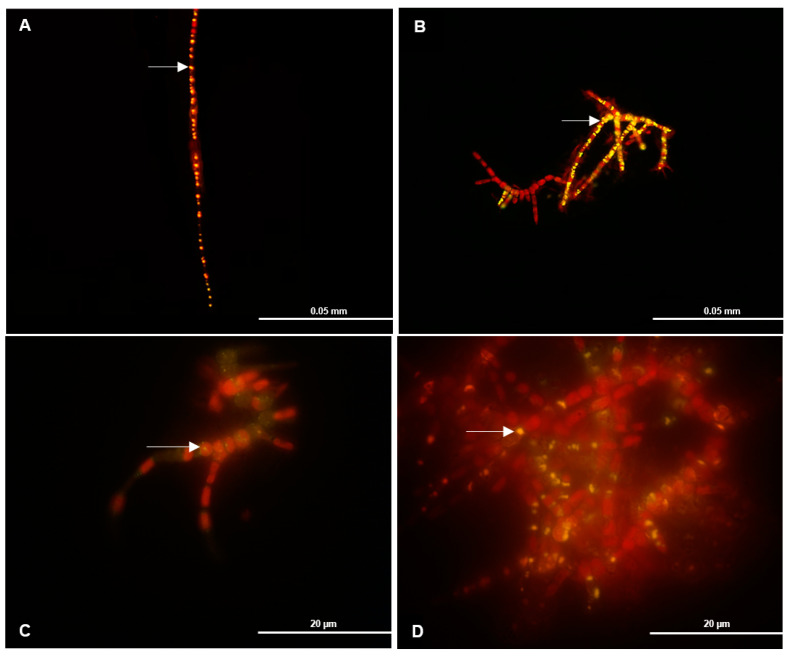
Polyhydroxybutyrate (PHB) in *Stigeoclonium* sp. B23 at day 6 (**A**,**C**) and day 12 (**B**,**D**) of cultivation. The arrows indicate where the yellow fluorescence was detected in the form of PHB granules after 12 days of cultivation. The PHB storage was not uniform in the filaments, but the highest amount of fluorescence was detected in no nitrate treatment; (**A**,**B**): no nitrate; (**C**,**D**): standard medium.

PHB arrangement in B23 was detected under fluorescence (Figure 4). For this experiment, standard and no nitrate media were the culture conditions at two different times. The Nile Red dye was directly added to the *Stigeoclonium* sp. B23 to stain colonies. A yellow fluorescence indicates the presence of PHB. Figure 4C shows small points of PHB in some filaments caused by the presence of nitrogen in the standard medium. Reversely, Figure 4D shows bigger points of PHB, and they are concentrated in various filaments. In this assay, in which a large incidence of fluorescence in BG-11 lacking nitrogen was observed, PHB storage was determined visually. Cultivation time was also a predominant factor in this result. While PHB production was low on the sixth day of cultivation (Figure 4A,C), 6 days later (Figure 4B,D), the period when microalga was about to reach the stationary phase, PHB increased accumulation.

### 3.5. PHB and Fluorescence Correlation

PHB was extracted from the lyophilized cell biomass using pure sodium hypochlorite with an active chlorine content of 2% to 2.5% *w/w*. The pellet was dissolved in hot chloroform and, finally, the biopolymer was hydrolyzed to crotonic acid. A curve of crotonic acid was assayed to quantify it (Figure S2).

**Figure 5:**
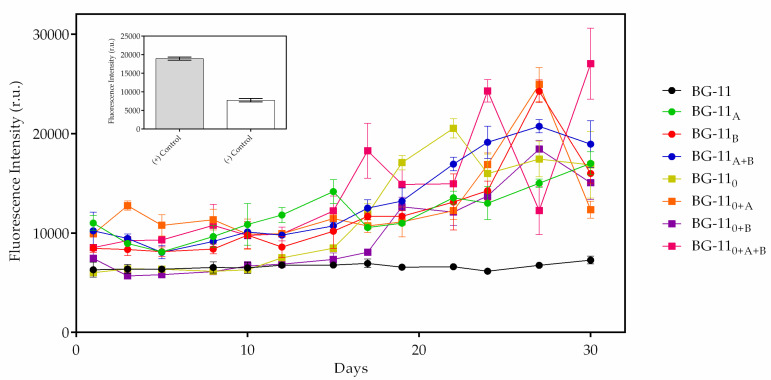
Quantitative fluorescence intensity of *Stigeoclonium* sp. B23 in eight modified growth conditions. Data are the mean ± SD of three replicates. (+) Control: crotonic acid, distilled water, and Nile Red, (−) Control: distilled water and Nile Red.

**Figure 6:**
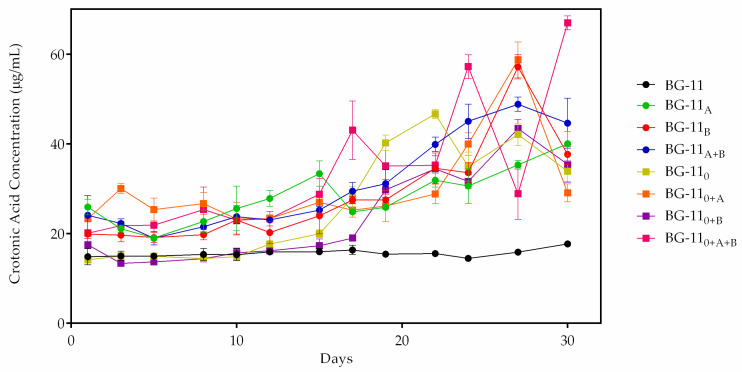
PHB in the form of crotonic acid of *Stigeoclonium* sp. B23 in 8 modified growth conditions. Data are the mean ± SD of three replicates.

In this work, the proportional increase of both fluorescence (Figure 5) and PHB (Figure 6) in the eight adjusted growth media were compared. An observation was made to see if there was an increase in the production of crotonic acid on the last day of cultivation, and it was compared with the first day of production. An ANOVA test proved to be significant in all source of variations of fluorescence (F = 18.0, *p* < 0.0001; Table S1) and PHB quantity (F = 24.62, *p* < 0.0001; Table S2), in which the differences in the quantity of both also have significant interactions between the growth period and different growth conditions. Tukey’s post hoc multiple comparisons test and statistical analyzes (Tables S3 and S4) showed highly significant PHB quantity when comparing all treatments (*p* < 0.0001), except for acetate and bicarbonate treatments (*p* > 0.99) and acetate plus bicarbonate compared to no nitrate plus acetate (*p* = 0.76). Furthermore, regarding the increase of fluorescence, acetate and bicarbonate supplementation proved to be significant when compared to the standard medium (*p* < 0.0001). However, treatment with acetate was not significant when compared to others such as bicarbonate, no nitrate, and no nitrate plus acetate (*p* = 0.99, 0.30, and 0.08, respectively). Additionally, this was observed on bicarbonate and nitrate-free treatment (*p* = 0.65) and acetate plus bicarbonate and no nitrate plus acetate treatment (*p* = 0.86). The fluorescence and PHB concentration data obtained are the mean ± SD of three replicates and are summarized in supplementary tables (Tables S5–S8).

**Figure 7:**
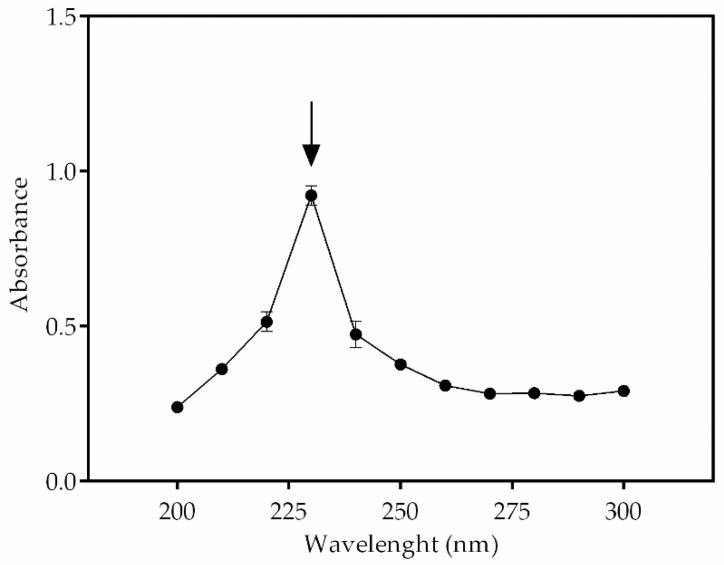
Occurrence of PHB in *Stigeoclonium* sp. B23 by absorbance spectrum. The black arrow indicates the wavelength between 225 and 235 nm, which confirms it as being PHB. Data are the mean ± SD of three replicates.

In the first four nitrate cultures, the presence of both sodium acetate and/or sodium bicarbonate was steadily and, over time, important for the production of crotonic acid. Standard medium showed no peaks of fluorescence or a difference of crotonic acid quantity during the 30 days of cultivation. In this treatment, fluorescence and crotonic acid values were 7284 ± 396.6 r.u./day and 17.7 ± 0.3 µg/mL/day; a 16.3% increased biopolymer. The sodium acetate treatment enhanced crotonic acid concentration. After a period of adaptation in the first 5 days, microalga started to accumulate on the biopolymer, with a slight decrease after day 15 of cultivation. This might be explained by the consumption of sodium acetate by microalga and the carbon sources becoming scarce. In this treatment, 40.1 ± 2.7 µg/mL/day of crotonic acid and 17,003.1 ± 1152.3 r.u./day of fluorescence were obtained. In addition, there was a 35.3% increase of crotonic acid in this treatment. Sodium bicarbonate induced crotonic acid production more than sodium acetate. In this treatment, 57.14 ± 2.6 µg/mL/day of crotonic acid and 24,259.6 ± 1140.8 r.u./day of fluorescence were quantified, which is a 65.1% increase in crotonic acid. When combining both supplements, increased PHB production was also observed. The maximum quantity of crotonic acid in this treatment was 48.8 ± 1.5 µg/mL/day; an increase of 50.6% of biopolymer. When it comes to nitrogen deprivation, biopolymer production was more effective with supplementation. With no nitrate treatments, 69.6% increased biopolymer was achieved, which corresponds to 46.6 ± 1,0 µg/mL/day of crotonic acid and 20,526 ± 978.2 r.u./day of fluorescence. Acetate treatment was vital to improve PHB production, with 58.7 ± 4.0 µg/mL/day of biopolymer and 24,933.6 ± 1698.4 r.u./day of fluorescence; a 60.2% increased biopolymer in this treatment. Inversely, bicarbonate was not efficient when compared with bicarbonate treatment. The concentration of crotonic acid was 43.4 ± 2.0 µg/mL/day, which corresponds to 18,431.3 ± 869.7 r.u./day; 59.6% increased PHB. When combining both supplements, crotonic acid production reached its highest concentration among all eight modified treatments, with 66.9 ± 1.5 µg/mL/day and 27,021 ± 3574.1 r.u./day fluorescence; 69.9% increased biopolymer. In addition, the absorbance spectrum suggested the type of biopolymer produced by microalga and showed a peak between 225 to 235 nm, which is reasonable to indicate the occurrence of PHB in *Stigeoclonium* sp. B23 (Figure 7).

## 4. Discussion

*Stigeoclonium* sp. B23 was cultivated in normal medium for a better understanding of its development under this condition. Quantities of cell biomass of chlorophyll *a* is a rapid and effective method to confirm cell viability without a lyophilized dry biomass requirement. The presence of nitrogen was central to maintain B23 growth, because BG-11 is a standard culture medium, which supported *Stigeoclonium* sp. B23 viability for long periods of time. This result is compared to those obtained of *Stigeoclonium* sp. LJ1 and *Stigeoclonium* sp. LJ2, which were cultivated with 46.2 mg/L of NO_3_-N on WC medium for 9 days to match the amount of nitrogen of horticultural wastewater [38]. *Stigeoclonium* sp. B23, however, was cultivated with 1.5 g/L of NaNO_3_ as the main source of nitrogen on BG-11 medium for 30 days, and this amount is about 30-fold higher than on the WC medium, suggesting that different species of Stigeoclonium can be cultivated in a short time with few nitrogen sources.

These findings demonstrate how *Stigeoclonium* sp. B23 can live in extreme environments, and, often, Stigeoclonium species dominate the cultivation of nitrogen and phosphorus in different proportions [39]. However, *Stigeoclonium* spp. was cultivated in various combinations with nitrogen and phosphorus concentrations and ratios to observe how this taxon responds to nutrient enrichment and, as a result, it was suggested that the microorganism responds to nutrient concentration, though the ratios were not that important [40]. It is reasonable to suggest that *Stigeoclonium* sp. B23 can survive with minimal quantities of nitrogen in order to produce sufficient biomass, along with high PHB yields, once the biopolymer is well-produced under this condition [11]. In the present work, we have suggested that there should be an increase in the production of biopolymers, even with a low amount of biomass in the complemented media. Figure 3 shows a difference in the quantity of chlorophyll, and this may be due to a decrease in the growth of microalgae, also in the complemented media. However, in these treatments, the development of PHB increased relative to the normal medium. Treatments with increased acetate and bicarbonate and without nitrate gave the highest concentration of PHB, while the treatment with only acetate gave the lowest amongst supplemented media. Inversely, these treatments did not exhibit increases in biomass production.

Not much is known about PHB production in eukaryotic microalgae. Bacteria and cyanobacteria are a well-studied group of PHB-producing microorganisms. [1,2,3,7]. Microalgae have emerged as a promising source of raw materials of lipids for biofuels, polyunsaturated fatty acids (PUFA), and, potentially, polyhydroxybutyrates. This is due to the rapid growth of cell biomass and easy cultivation, requiring small quantities of nutrients for intracellular bioaccumulation of metabolites [7]. PHB arrangement in *Stigeoclonium* sp. B23 was detected under fluorescence (Figure 4). For this experiment, BG-11 (Figure 4C,D) and BG-11_0_ (Figure 4A,B) were the culture conditions. The presence of PHB in both BG-11 treatments was suggested by yellow fluorescence, but a high incidence of BG-11 fluorescence without nitrogen was also observed. Nutritional deficiency caused extreme stress in *Stigeoclonium* sp. B23 for PHB synthesis, showing the importance of this process in the production and storage of biopolymers. In addition, PHB visualization occurred during the whole process of cultivation, but lots of fluorescence was detected as soon as B23 reached the stationary phase (precisely in the log phase), in which the microalga started to grow exponentially in BG-11. It confirms that PHB production can occur at any stage of growth [21]. The biopolymer synthesis was not homogeneous and did not occur in the entire *Stigeoclonium* sp. B23 filaments, even though all cells were under the same culture conditions; not all cells were capable of storing the biopolymer or did not require it. Biochemically, once cyanobacteria are cultivated under standard conditions, i.e., BG-11 medium, NADPH in Tricarboxylic Acid Cycle (TCA) remains constant throughout the cell culture period, and the production of NADPH at this time does not vary significantly. On the other hand, under nitrogen deprivation, when there is an increase of PHB production, the consumption of NADPH by cells decreases due to the limitation of nitrogen sources blocking the metabolic pathway of amino acid synthesis, which is the reaction of α-ketoglutarate to glutamate, resulting in an accumulation of NADPH in intracellular compartments. This excess of NADPH may be responsible for PHB production in nitrogen-deficient cells [41]. *Stigeoclonium* sp. B23 adapted to environmental conditions under nitrogen depleted, maximized its metabolism to different phenotypic forms, and the microalga required carbon sources for later storage in the form of PHB granules. *Stigeoclonium* sp. B23 was under total nitrogen deprivation from the first day of cultivation and, initially, nutritional stress did not increase the fluorescence up to about half of the cultivation period in all conditions. This can be demonstrated by the delay of *Stigeoclonium* sp. B23 of treating stress by reorganizing its metabolism due to a lack of concentration of nitrogen and control of the cellular storage of carbon in the form of PHB [22,40,42]. In addition, it is rational to assume that the output of PHB did not increase exponentially during the increasing period in *Stigeoclonium* sp. B23. Production, consumption, and increased production of the carbon reserve were observed after the first consumption. Stress caused by a lack of nitrogen and a lack of microalgae growth in optimized treatments can cause the long-term consumption of previously stored PHB (Figure 5 and Figure 6). This was also observed in treatments such as nitrate-free, processing, storage and subsequent consumption of polymer due to nutritional deprivation and the need for carbon sources for osmotic control, and defense against cell desiccation of microalgae and ultraviolet rays, which are the direct consequences of long periods of nutritional deficiency and malfunction of photosynthesis [3]. Besides that, the utilization of sodium acetate and sodium bicarbonate as carbon sources for PHB production are in accordance with the study proposed by Higuchi-Takeuchi et al. [43], in which 12 sulfurous bacteria were reported in producing PHB under limited conditions of depleted nitrogen and in the presence of both chemicals.

Nile Red is a fluorescent dye soluble in neutral lipids and a range of polyhydroxybutyrates. High fluorescence intensity in organic solvents can be detected, and it is highly hydrophobic [26]. Crotonic acid is unsaturated carboxylic acid and it can be obtained by several methods such as the oxidation of crotonaldehyde, condensation of acetaldehyde, and by alkaline hydrolysis of allyl cyanide, and it is formed by the distillation of 3-hydroxybutyric acid, which is the monomer of poly(3-hydroxybutyrate), also known as P(3HB). Crotonic acid is the major product from the thermal degradation process of PHB of β-elimination [44]. Dye is commonly used as a fluorophore to assess the polarity of these solvents and can be quantified by its strength as a result of PHB accumulation in the form of its monomeric crotonic acid, which provides a qualitative and quantitative indirect indicator [26,27,45,46]. The fluorescence intensity is correlated to PHB concentration and offers a fast and appropriate response [11]. In our study, the correlation was done in triplicate. BG-11 is a standard medium commonly used for microalgae and cyanobacteria cultivation. However, carbon supplementation is a suitable method for offering more nutrients for biomass and PHB enrichment. Moreover, microalgae absorb carbon from inorganic sources such as carbon dioxide by the photosynthesis process [47].

It was noted that the quantification of PHB occurred on the final day of cultivation (Day 30), as suggested by the methodology, in which long periods of cultivation could lead to a large accumulation of the biopolymer and thus achieve its maximum PHB productivity values. The quantification of PHB in BG-11 was performed during the end of the stationary phase of B23, a period when microalga reached its maximum growth and the biomass volume was high compared to no nitrate treatment, which was impossible to cultivate for long periods but, inversely, microalga produced more biopolymer under this condition.

The PHB extraction methodology used in this work is widely used and is one of the most efficient techniques in extracting and quantifying PHB because it is fast, has low cost, and is accurate. This method was tested and documented on the development of PHB in 10 bacterial species and verified that *Bacillus* spp. was detected to be best PHB producer [37]. Optimization for PHB enrichment was assayed using different carbon and nitrogen sources, as well as the alteration of parameters such as temperature and pH. As a result, when the strains were cultivated with glucose as a carbon source and peptone as a source of nitrogen, PHB quantities were 6.1 ± 0.07 g/L and 5.2 ± 0.33 g/L, respectively. In the same way, 5.202 g/L of PHB was obtained as the maximum quantity in *Bacillus* sp. NA10 [48], indicating that the cultivation methodology proposed in this work was more effective using only BG-11 as a carbon source and nitrogen deprivation as nutritional stress to obtain a greater enrichment in the production of the biopolymer. It is the easiest way to accumulate a biopolymer.

Even though BG-11 itself is a poor medium for PHB production, the quantity of crotonic acid obtained can be explained because of the cultivation time of *Stigeoclonium* sp. B23 and the subsequent consumption and absence of sodium nitrate. BG-11 is a rich phosphate, sulfate, and metals medium. Sodium nitrate itself inhibited the production of PHB in a normal medium without supplementation. *Stigeoclonium* sp. B23 will live with a minimum amount of nitrogen to provide biomass for its growth [11]. The only carbon source of BG-11 is provided by sodium carbonate at the final concentration of 0.02 g/L. This quantity is a low amount for inducing biomass production and biotechnological products, and is insufficient to increase PHB accumulation. However, salts such as sodium acetate enriched PHB production and could easily optimize it more than standard medium alone [49]. Besides that, microalga was cultivated for 30 days under 25 °C and controlled light. These parameters are important for the photosynthesis process, which is another source of carbon for the development of PHB [50]. The input of substrates such as sodium acetate and/or bicarbonate for optimization usually does not cause problems. Under photosynthesis, CO_2_ alone is used as the sole carbon source, but the carbon in aqueous solution is low and CO_2_ fixation is a deficient method. To overcome this, biochemical and engineering processes have emerged to improve the production of biotechnological compounds [51].

BG-11 cultures demonstrated that sodium bicarbonate had a significant effect on the development of PHB and was more successful than sodium acetate, mainly due to the ability of sodium bicarbonate to release CO_2_ when reacting with acid and the continuous absorption of it by microalgae to store the biopolymer and increase biomass. Our results suggest how second carbon sources increase the accumulation of PHB and how *Stigeoclonium* sp. B23 is able to accumulate it under different culture conditions at different growth stages with minimal quantities of salts. This was confirmed by the increased production of the biopolymer during the 30 days of cultivation of all assays, except BG-11. However, sodium acetate could not increase PHB production. The key carbon source for PHB enrichment was sodium bicarbonate treatment. The latest scientific reports concentrate on bacteria and cyanobacteria in the development, identification, and characterization of a variety of PHBs. It is a well-known and written biopolymer in this group. The present work suggested a new alternative to the development of PHB from Amazonian filamentous microalgae. There are few studies published in the literature for the development of biopolymers in this category of eukaryotic microalgae. Optimization of the development of PHB can lead to an increase in the concentration of biopolymers compared to bacterial species. High yields of PHB can be achieved using species from the genus Stigeoclonium, as shown in the results section. Microalgae are capable of intracellularly accumulating PHB granules, as discussed above. However, different behaviors are observed through optimization, and this might be explained by various microalgae sources, nutritional concentrations, and growth conditions.

## 5. Conclusions

The findings of the present work have confirmed that *Stigeoclonium* sp. B23 was able to generate and store polyhydroxybutyrate under different culture conditions by intracellular storage of biopolymers under complete nitrogen deprivation and with limited amounts of sodium acetate and sodium bicarbonate. In addition, the development of PHB was more important in the sense of total nitrogen deficiency, as shown by the statistical study, by the fluorescence quantification of the biopolymer itself. This work is the first to record the synthesis of PHB in Amazonian microalgae and the discovery of biopolymer storage on a species of the genus Stigeoclonium after optimization. Microalgae have been isolated from a poorly explored and studied area and have been shown to be active in comparison to other cyanobacterial and bacterial species and may potentially be used for large-scale biopolymer development. This shows the great potential of Amazon microalgae biodiversity combined with biotechnological research for promoting technological and social growth in the Amazon region.

## Figures and Tables

**Table 1:** Treatments of *Stigeoclonium* sp. B23 in different BG-11 media.

Treatment	Carbon Source 1	Carbon Source 2	Nitrogen Source
BG-11	*	*	Sodium nitrate
BG-11_A_	Sodium acetate	*	Sodium nitrate
BG-11_B_	*	Sodium bicarbonate	Sodium nitrate
BG-11_A+B_	Sodium acetate	Sodium bicarbonate	Sodium nitrate
BG-11_0_	*	*	*
BG-11_0+A_	Sodium acetate	*	*
BG-11_0+B_	*	Sodium bicarbonate	*
BG-11_0+A+B_	Sodium acetate	Sodium bicarbonate	*

* Carbon or nitrogen deficiency.

## Supplementary Materials

The following are available online at https://www.mdpi.com/2218-273X/10/12/1628/s1, Figure S1. 18S rRNA sequence from Stigeoclonium sp. B23. Figure S2. Standard curve from commercial acid crotonic. UV absorbance 235 nm is plotted as a function of amount of acid crotonic in μg/mL. Data are the mean±SD of three replicates. Table S1. ANOVA table of fluorescence of PHB quantity in modified BG-11 media of Stigeoclonium sp. B23 at the 95% confidence level. Table S2. ANOVA table of PHB quantity in modified BG-11 media of Stigeoclonium sp. B23 at the 95% confidence level. Table S3. Tukey’s post hoc multiple comparisons of fluorescence of PHB between modified BG11 media. The mean difference is significant at the 0.05 level. Table S4. Tukey’s post hoc multiple comparisons of PHB between modified BG-11 media. The mean difference is significant at the 0.05 level. Table S5. Data of mean and standard deviation of fluorescence in modified growth media with nitrate. Table S6. Data of mean and standard deviation of fluorescence in modified growth media with nitrogen deprivation. Table S7. Data of mean and standard deviation of extraction-based PHB quantification in modified growth media with nitrogen. Table S8. Data of mean and standard deviation of extraction-based PHB quantification in modified growth media without nitrogen.
