# Supplementary material for: Optimization of Polyhydroxybutyrate Production by Amazonian Microalga Stigeoclonium sp. B23
Source: Biomolecules. 2020 Dec 3;10(12):1628. doi: 10.3390/biom10121628 (PMC7761742; doi:10.3390/biom10121628)
Supplement: Supplementary file 1 [file biomolecules-10-01628-s001.pdf]

Supplementary Materials: The following are available online at https://www.mdpi.com/2218-273X/10/12/1628/s1.

TCAGGTGGGAGGGTTTAATGAACTTCTCGGGGACGGTGGGAGAGACTCCCGACCGTCCTCAATCC
GAACACTTCACCAGCACACCCAATCGGTAGGAGCGACGGGCGGTGTGTACAAAGGGCAGGGACG
TAATCAACGCGAGCTGATGACTCGCGCTTACTAGGCATTCCTCGTTGAAGACTAATAATTGCAATA
ATCTATCCCCATCACGATGCAGTTTCAAAGATTACCCGGGCCTCTCGGCCAAGGATAGGCTCGTTG
AATGCATCAGTGTAGCGCGCGTGCGGCCCAGAACATCTAAGGGCATCACAGACCTGTTATTGCCT
CATACTTCCATTGCCTAAACGACAATAGTCCCTCTAAGAAGTCTGCCAGCCGCAAGAAGCGGCAG
TGACTATTTAGCAGGCTGAGGTCTCGTTCGTTACCGGAATCAACCTGACAAGGCAACCCACCAAC
TAAGAACGGCCATGCACCACCACCCATAGAATCAAGAAAGAGCTCTCAATCTGGATCA

Figure S1. 18S rRNA sequence from *Stigeoclonium* sp. B23.

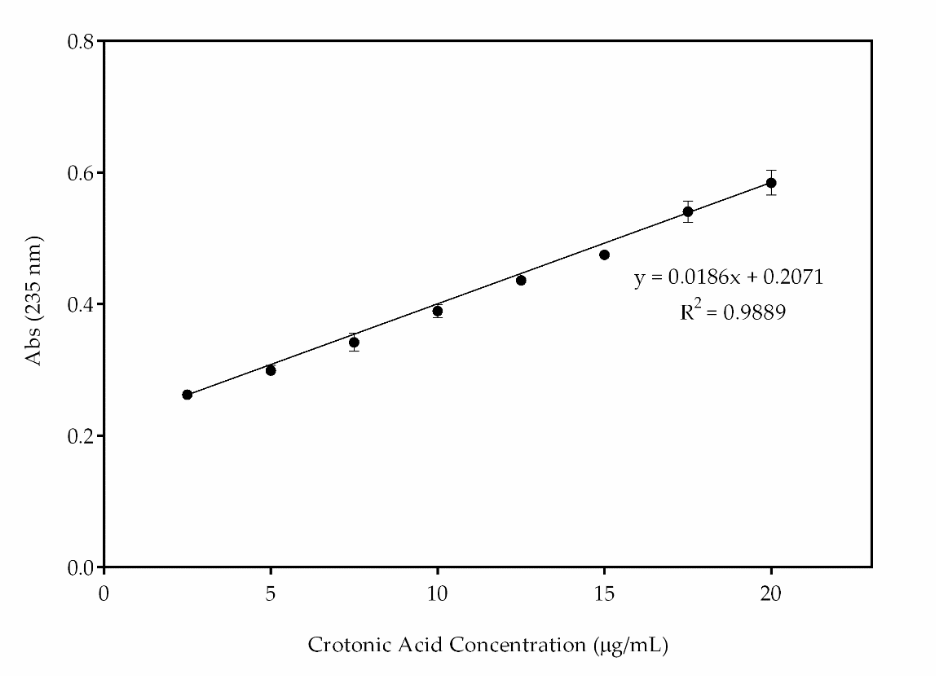

Figure S2. Standard curve from commercial acid crotonic. UV absorbance 235 nm is plotted as a function of amount of acid crotonic in µg/mL. Data are the mean±SD of three replicates.

Table S1. ANOVA table of fluorescence of PHB quantity in modified BG-11 media of *Stigeoclonium* sp. B23 at the 95% confidence level.

	**Degrees of freedom**	**Sum of squares**	**Mean of squares**	**Fcalc**	**P value**
Interaction	84	1.971e+9	2.346e+7	18.00	P < 0.0001
Daily fluorescence	12	3.281e+9	2.734e+8	209.8	P < 0.0001
Treatments	7	1.437e+9	2.052e+8	157.5	P < 0.0001
Residual (error)	208	2.711e+8	1.303e+6		
Total	311	6.959e+9	-		

Table S2. ANOVA table of PHB quantity in modified BG-11 media of *Stigeoclonium* sp. B23 at the 95% confidence level.

	Degrees of freedom	Sum of squares	Mean of squares	Fcalc	P value
Interaction	84	11,252	134.0	24.62	P < 0.0001
Daily PHB	12	18,276	1,523	279.9	P < 0.0001
Treatments	7	7,993	1,142	209.9	P < 0.0001
Residual (error)	208	1,132	5.441		
Total	311	38,653	-		

Table S3. Tukey's post hoc multiple comparisons of fluorescence of PHB between modified BG-11 media. The mean difference is significant at the 0.05 level.

Treatment comparisons	Mean difference	95% confidence interval		P value
		Lower bound	Upper bound	
BG-11 vs. BG-11$_{A}$	-5285	-6076	-4493	< 0.0001
BG-11 vs. BG-11$_{B}$	-5146	-5938	-4355	< 0.0001
BG-11 vs. BG-11$_{A+B}$	-6386	-7178	-5594	< 0.0001
BG-11 vs. BG-11$_{0}$	-4695	-5487	-3904	< 0.0001
BG-11 vs. BG-11$_{0+A}$	-6026	-6818	-5235	< 0.0001
BG-11 vs. BG-11$_{0+B}$	-3080	-3871	-2288	< 0.0001
BG-11 vs. BG-11$_{0+A+B}$	-7345	-8136	-6553	< 0.0001
BG-11$_{A}$ vs. BG-11$_{B}$	138.5	-653.1	930.1	0.9995
BG-11$_{A}$ vs. BG-11$_{A+B}$	-1101	-1893	-309.7	0.0008
BG-11$_{A}$ vs. BG-11$_{0}$	589.3	-202.3	1381	0.3099
BG-11$_{A}$ vs. BG-11$_{0+A}$	-741.7	-1533	49.92	0.0844
BG-11$_{A}$ vs. BG-11$_{0+B}$	2205	1413	2997	< 0.0001
BG-11$_{A}$ vs. BG-11$_{0+A+B}$	-2060	-2852	-1269	< 0.0001
BG-11$_{B}$ vs. BG-11$_{A+B}$	-1240	-2031	-448.2	< 0.0001
BG-11$_{B}$ vs. BG-11$_{0}$	450.8	-340.8	1242	0.6586
BG-11$_{B}$ vs. BG-11$_{0+A}$	-880.2	-1672	-88.60	0.0178
BG-11$_{B}$ vs. BG-11$_{0+B}$	2066	1275	2858	< 0.0001
BG-11$_{B}$ vs. BG-11$_{0+A+B}$	-2199	-2990	-1407	< 0.0001
BG-11$_{A+B}$ vs. BG-11$_{0}$	1691	899.0	2482	< 0.0001
BG-11$_{A+B}$ vs. BG-11$_{0+A}$	359.6	-432.0	1151	0.8605
BG-11$_{A+B}$ vs. BG-11$_{0+B}$	3306	2515	4098	< 0.0001
BG-11$_{A+B}$ vs. BG-11$_{0+A+B}$	-958.8	-1750	-167.2	0.0064
BG-11$_{0}$ vs. BG-11$_{0+A}$	-1331	-2123	-539.4	< 0.0001
BG-11$_{0}$ vs. BG-11$_{0+B}$	1616	824.1	2407	< 0.0001
BG-11$_{0}$ vs. BG-11$_{0+A+B}$	-2649	-3441	-1858	< 0.0001
BG-11$_{0+A}$ vs. BG-11$_{0+B}$	2947	2155	3738	< 0.0001
BG-11$_{0+A}$ vs. BG-11$_{0+A+B}$	-1318	-2110	-526.9	< 0.0001
BG-11$_{0+B}$ vs. BG-11$_{0+A+B}$	-4265	-5057	-3474	< 0.0001

Table S4. Tukey's post hoc multiple comparisons of PHB between modified BG-11 media. The mean difference is significant at the 0.05 level.

Treatment comparisons	Mean difference	95% confidence interval		P value
		Lower bound	Upper bound	
BG-11 vs. BG-11$_{A}$	-12.40	-14.02	-10.79	< 0.0001
BG-11 vs. BG-11$_{B}$	-12.36	-13.98	-10.74	< 0.0001
BG-11 vs. BG-11$_{A+B}$	-15.00	-16.62	-13.38	< 0.0001
BG-11 vs. BG-11$_{0}$	-10.32	-11.94	-8.700	< 0.0001
BG-11 vs. BG-11$_{0+A}$	-14.17	-15.78	-12.55	< 0.0001
BG-11 vs. BG-11$_{0+B}$	-7.614	-9.231	-5.996	< 0.0001
BG-11 vs. BG-11$_{0+A+B}$	-17.51	-19.13	-15.90	< 0.0001
BG-11$_{A}$ vs. BG-11$_{B}$	0.04420	-1.573	1.662	> 0.9999
BG-11$_{A}$ vs. BG-11$_{A+B}$	-2.594	-4.212	-0.9766	< 0.0001
BG-11$_{A}$ vs. BG-11$_{0}$	2.087	0.4692	3.704	0.0027
BG-11$_{A}$ vs. BG-11$_{0+A}$	-1.762	-3.379	-0.1443	0.0222
BG-11$_{A}$ vs. BG-11$_{0+B}$	4.791	3.173	6.408	< 0.0001
BG-11$_{A}$ vs. BG-11$_{0+A+B}$	-5.109	-6.726	-3.491	< 0.0001
BG-11$_{B}$ vs. BG-11$_{A+B}$	-2.638	-4.256	-1.021	< 0.0001
BG-11$_{B}$ vs. BG-11$_{0}$	2.043	0.4250	3.660	0.0036
BG-11$_{B}$ vs. BG-11$_{0+A}$	-1.806	-3.424	-0.1885	0.0170
BG-11$_{B}$ vs. BG-11$_{0+B}$	4.747	3.129	6.364	< 0.0001
BG-11$_{B}$ vs. BG-11$_{0+A+B}$	-5.153	-6.771	-3.536	< 0.0001
BG-11$_{A+B}$ vs. BG-11$_{0}$	4.681	3.063	6.298	< 0.0001
BG-11$_{A+B}$ vs. BG-11$_{0+A}$	0.8323	-0.7852	2.450	0.7644
BG-11$_{A+B}$ vs. BG-11$_{0+B}$	7.385	5.767	9.002	< 0.0001
BG-11$_{A+B}$ vs. BG-11$_{0+A+B}$	-2.515	-4.132	-0.8974	< 0.0001
BG-11$_{0}$ vs. BG-11$_{0+A}$	-3.849	-5.466	-2.231	< 0.0001
BG-11$_{0}$ vs. BG-11$_{0+B}$	2.704	1.087	4.322	< 0.0001
BG-11$_{0}$ vs. BG-11$_{0+A+B}$	-7.196	-8.813	-5.578	< 0.0001
BG-11$_{0+A}$ vs. BG-11$_{0+B}$	6.553	4.935	8.170	< 0.0001
BG-11$_{0+A}$ vs. BG-11$_{0+A+B}$	-3.347	-4.965	-1.730	< 0.0001
BG-11$_{0+B}$ vs. BG-11$_{0+A+B}$	-9.900	-11.52	-8.282	< 0.0001

Table S5. Data of mean and standard deviation of fluorescence in modified growth media with nitrate.

Day	BG-11		$BG\text{-}11_A$		$BG\text{-}11_B$		$BG\text{-}11_{A+B}$	
	Mean	SD	Mean	SD	Mean	SD	Mean	SD
1	6310.667	759.2953	11013.330	730.847	8455.333	414.9811	10232.000	1862.828
3	6387.333	434.7382	8957.333	335.287	8350.667	629.5438	9463.333	429.1449
5	6372.000	248.3626	8094.000	300.020	8124.667	477.6037	8062.000	639.9781
8	6533.333	568.0998	9650.667	1082.930	8394.667	487.1194	9154.667	644.3301
10	6502.000	567.757	10868.000	2102.076	9788.667	1353.333	10108.000	633.0593
12	6768.000	167.7975	11817.330	749.4113	8595.333	1053.186	9792.667	21.00793
15	6789.333	314.454	14167.000	1194.420	10182.670	1717.009	10719.330	354.0301
17	6950.667	436.0566	10553.330	32.08323	11668.670	352.8701	12512.670	826.8067
19	6564.000	124.3865	10992.000	230.7293	11688.670	545.3158	13222.330	1310.249
22	6612.667	139.7331	13555.000	656.6285	13121.000	2820.188	16935.000	687.5551
24	6159.333	110.1514	13007.670	1660.981	14261.330	956.0807	19125.670	1627.110
27	6750.667	236.7474	15005.670	411.4564	24259.670	1140.881	20732.670	668.7431
30	7284.000	396.651	17003.000	1152.361	15992.330	978.2624	18941.330	2360.497

Table S6. Data of mean and standard deviation of fluorescence in modified growth media with nitrogen deprivation.

Day	$BG\text{-}11_0$		$BG\text{-}11_{0+A}$		$BG\text{-}11_{0+B}$		$BG\text{-}11_{0+A+B}$	
	Mean	SD	Mean	SD	Mean	SD	Mean	SD
1	6021.333	212.5308	9906.000	307.5906	7444.667	1393.063	8537.333	82.80902
3	6388.667	192.2533	12762.000	456.6487	5688.667	306.8376	9250.000	414.8445
5	6363.333	306.0349	10773.330	1077.869	5820.667	251.0166	9301.333	1246.044
8	6146.667	251.7962	11342.000	1042.466	6125.333	246.4332	10768.670	2122.887
10	6316.000	191.8228	9886.000	1528.318	6684.000	442.7144	9769.333	356.0019
12	7506.667	209.0391	9934.000	326.2147	6877.333	169.3084	9909.333	692.9166
15	8478.000	366.6443	11461.330	259.556	7346.667	648.9448	12222.670	1481.568
17	11925.330	999.2043	10717.330	662.143	8078.667	236.5784	18272.670	2760.394
19	17077.330	724.7899	11115.330	1495.115	12634.000	955.7155	14900.000	2195.070
22	20526.330	978.2159	12254.670	911.7156	12102.330	1325.480	14958.330	319.4391
24	15975.670	1952.396	16895.330	1155.858	13733.330	360.1852	24287.000	1128.177
27	17432.000	1791.150	24933.670	1698.459	18431.330	869.7151	12269.000	2430.500
30	16866.000	3338.855	12345.330	846.0693	15052.670	1690.447	27021.000	3574.167

Table S7. Data of mean and standard deviation of extraction-based PHB quantification in modified growth media with nitrogen.

Day	BG-11		$BG\text{-}11_A$		$BG\text{-}11_B$		$BG\text{-}11_{A+B}$	
	**Mean**	**SD**	**Mean**	**SD**	**Mean**	**SD**	**Mean**	**SD**
1	14.86196	1.78843	25.93852	1.721422	19.91346	0.9774411	24.09818	4.387668
3	15.04253	1.023973	21.09586	0.7897311	19.66693	1.482814	22.28768	1.010799
5	15.00642	0.5849894	19.06238	0.7066602	19.13461	1.124939	18.98701	1.507392
8	15.38642	1.338091	22.72892	2.550712	19.77057	1.147349	21.56065	1.517645
10	15.31262	1.337283	25.59621	4.951184	23.05397	3.187614	23.80612	1.491093
12	15.93915	0.3952259	27.83225	1.765149	20.24322	2.480652	23.06339	0.04948387
15	15.9894	0.7406616	33.3666	2.813316	23.98199	4.04421	25.24604	0.8338712
17	16.3694	1.027077	24.85505	0.07557172	27.48208	0.8311429	29.47002	1.947445
19	15.45865	0.292976	25.88827	0.5434573	27.52919	1.284424	31.14155	3.086132
22	15.57328	0.3291237	31.92511	1.546607	34.56954	3.428161	39.8863	1.619455
24	14.50551	0.2594465	30.63593	3.912241	33.58879	2.251937	45.04615	3.832462
27	15.89832	0.557632	35.34198	0.9691374	57.13867	2.687207	48.83124	1.575145
30	17.70924	0.3660937	40.04646	2.714248	37.66596	2.304179	44.61197	5.559862

Table S8. Data of mean and standard deviation of extraction-based PHB quantification in modified growth media without nitrogen.

Day	$BG\text{-}11_0$		$BG\text{-}11_{0+A}$		$BG\text{-}11_{0+B}$		$BG\text{-}11_{0+A+B}$	
	**Mean**	**SD**	**Mean**	**SD**	**Mean**	**SD**	**Mean**	**SD**
1	14.18046	0.5005932	23.33033	0.7244924	17.53296	3.281191	20.1066	0.195045
3	15.04568	0.4528322	30.0573	1.075581	13.39691	0.7227196	21.7852	0.9771153
5	14.98601	0.7208246	25.37323	2.538794	13.70782	0.5912426	21.90611	2.934909
8	14.47567	0.5930778	26.71265	2.455403	14.42543	0.5804445	25.36224	5.000205
10	14.87452	0.4518149	23.28322	3.599769	15.7413	1.042759	23.00843	0.8385173
12	17.67899	0.4923672	23.39628	0.7683578	16.19667	0.3987814	23.33818	1.632085
15	19.96685	0.8635891	26.99373	0.6113493	17.30213	1.528513	28.78696	3.489658
17	28.08663	2.353507	25.24133	1.559597	19.02627	0.5572321	43.03701	6.501774
19	40.22154	1.707151	26.17877	3.521567	29.75581	2.251072	35.09309	5.170225
22	46.67859	1.018235	28.86233	2.147433	34.45121	2.871845	35.2305	0.7523999
24	34.96004	3.216693	39.98569	2.492167	31.63439	1.517311	57.20304	2.657284
27	42.10924	2.514637	58.72619	4.000516	43.41073	2.048505	28.8961	5.724751
30	33.92219	2.247277	29.07589	1.992817	35.45268	3.981642	66.97599	1.546686
